# Review of Short-Wavelength Infrared Flip-Chip Bump Bonding Process Technology

**DOI:** 10.3390/s25010263

**Published:** 2025-01-05

**Authors:** Junhao Du, Xuewei Zhao, Jiale Su, Ben Li, Xiangliang Duan, Tianyu Dong, Hongxiao Lin, Yuhui Ren, Yuanhao Miao, Henry H. Radamson

**Affiliations:** 1 Research and Development Center of Optoelectronic Hybrid IC, Guangdong Greater Bay Area Institute of Integrated Circuit and System, Guangzhou 510535, China; 2 Key Laboratory of Microelectronic Devices & Integrated Technology, Institute of Microelectronics, Chinese Academy of Sciences, Beijing 100029, China

**Keywords:** Ge, InGaAs, SWIR, flip chip, FPAs

## Abstract

Short-wave infrared (SWIR) imaging has a wide range of applications in civil and military fields. Over the past two decades, significant efforts have been devoted to developing high-resolution, high-sensitivity, and cost-effective SWIR sensors covering the spectral range from 0.9 μm to 3 μm. These advancements stimulate new prospects across a wide array of fields including life sciences, medical diagnostics, defense, surveillance, security, free-space optics (FSO), thermography, agriculture, food inspection, and LiDAR applications. In this review, we begin by introducing monolithic SWIR image sensors and hybrid SWIR image sensors and indicate that flip-chip bump bonding technology remains the predominant integration method for hybrid SWIR image sensors owing to its outstanding performance, adaptable integration with innovative epitaxial SWIR materials, long-term stability, and long-term reliability. Subsequently, we comprehensively summarize recent advancements in epitaxial thin-film SWIR sensors, encompassing FPAs and flip-chip bump bonding technology for epitaxial InGaAs and Ge (Sn) thin-film SWIR sensors. Finally, a summary and outlook regarding the development of InGaAs and Ge (Sn) SWIR sensors are provided and discussed. The ongoing evolution of epitaxial thin-film SWIR sensors with flip-chip bump bonding technology is poised to foster new applications in both academic and industry fields.

## 1. Introduction

Infrared imagers are classified into near-infrared (NIR) imagers (0.75–1 μm), short-wave infrared (SWIR) imagers (1–3 μm), mid-infrared (MIR) imagers (3–5 μm), and long-wave infrared (LWIR) imagers (8–14 μm) [1,2]. Compared to other infrared imagers, SWIR imagers hold the advantages of similarity to visible light imaging technology, enhanced contrast and visibility, improved penetration ability, higher sensitivity, reduced power consumption, wider bandwidth options, and potential CMOS compatibility, thereby making SWIR imagers an attractive choice for a variety of applications [3,4,5]. SWIR imagers typically consist of several key components, such as SWIR focal plane arrays (FPAs), an optics system, CMOS readout integrated circuits (ROICs), mechanical components, control electronics, etc. Among these components, SWIR FPAs and CMOS ROICs play crucial roles in SWaP imagers [6,7,8]. To achieve high-performance SWIR imagers, SWIR FPAs need to continuously improve their miniaturization and integration levels [9,10]. Based on the integration method, SWIR imaging sensors are divided into monolithic SWIR image sensors and hybrid SWIR image sensors [11,12].

The monolithic SWIR image sensor refers to a sensor that integrates SWIR FPAs directly onto a single semiconductor substrate, which offers several advantages over hybrid sensors, including potentially lower manufacturing costs and simplified fabrication processes [13,14,15,16]. Additionally, monolithic SWIR image sensors can achieve higher levels of integration and miniaturization [17,18], making them well suited for compact and portable imaging devices [19]. To date, emerging lead sulfide [20,21,22] (PbS) and mercury telluride (HgTe) colloidal quantum dot [23,24,25,26] (CQD) SWIR sensors adopt the monolithic integration scheme, where the FPAs are directly stacked onto CMOS ROICs [27]. The pixel size, pixel pitch, and array specifications are defined by the CMOS ROICs [28,29]. The monolithic integrated CQD sensor exhibits high yield, providing a low-cost solution for high-resolution SWIR imaging technology [30]. However, CQD sensors have several disadvantages: they contain toxic Pb and Hg elements [31,32], which prohibit their use in consumer electronic products; it is extremely challenging to control the nanoscale size [33], uniform distribution [34], and batch-to-batch consistency of PbS CQDs [35]; they have lower carrier mobility [36], making them unsuitable for avalanche-detection chips; they are incompatible with CMOS manufacturing processes; and they have a lower signal-to-noise ratio due to higher dark currents [37], lower average external quantum efficiency (EQE) [36,38,39], a slower response speed [40], and poorer long-term stability [22].

Unlike monolithic SWIR image sensors, hybrid SWIR image sensors utilize flip-chip bump bonding technology to integrate SWIR FPAs with CMOS ROICs [41,42], which was commonly employed in non-Si epitaxial thin-film SWIR sensors, such as mercury cadmium telluride [43,44,45] (HgCdTe, MCT), antimonide-based type II superlattices [46,47,48] (Sb-based T2SLs), InGaAs [2,49], Ge (Sn) [50,51,52,53,54], etc. However, the hybrid SWIR image sensor exhibits several drawbacks and limitations in its manufacturing cost, process complexity, size and weight constraints, reliability issues, performance limitations, technological maturity challenges, and limited customization level [22]. The pixel size, pixel pitch, and array specifications of FPAs are limited by the complex and expensive flip-chip bump bonding technology [11,55,56]. Specifically, the size of the InGaAs bump limits the pixel pitch, with the latest reported result being 5 μm. Although Sony Company has proposed Cu-Cu bonding to reduce the pixel pitch of the InGaAs SWIR image sensor to 5 μm, the specifications of FPAs are still at least an order of magnitude lower than those of CMOS sensors [57,58]. Despite these limitations, flip-chip bump bonding technology remains the mainstream integration scheme for forming SWIR image sensors. Continued advancements in flip-chip processing are also highly expected to achieve high-resolution SWIR sensors in the future, which is mainly attributed to the flip chip’s excellent performance, especially in terms of responsivity and quantum efficiency (QE), its flexible integration structure with novel SWIR materials, and its long-term stability and long-term reliability [11,56].

In this work, we review the progress in epitaxial thin-film SWIR sensors over the past two decades, focusing on FPAs and flip-chip bump bonding technology for epitaxial InGaAs and Ge (Sn) thin-film SWIR sensors. The structure of this review is as follows. In Section 2, we discuss the current status of epitaxial InGaAs and Ge (Sn) thin-film FPAs. Section 3 presents and discusses the recent progress in the mainstream flip-chip bump bonding technology used for integrating SWIR FPAs. Section 4 provides a summary and outlook regarding the development of InGaAs and Ge (Sn) SWIR sensors.

## 2. Epitaxial InGaAs and Ge (Sn) Thin Film SWIR FPAS

Epitaxy and crystal quality optimization of InGaAs and Ge (Sn) thin films are critical for SWIR FPAs, which has great significance for the hybrid SWIR image sensor industry. Researchers have tried to optimize the crystal quality, doping concentration, and surface morphology of InGaAs and Ge (Sn) films by precisely controlling epitaxial growth conditions, including, growth temperature, gas flow rate, and deposition pressure. With the continuous developments in hybrid SWIR image sensor technology, the market has introduced a group of stricter requirements in terms of pixel size, pixel pitch, array specifications, spectral response range, peak responsivity, and peak quantum efficiency, etc. Consequently, the epitaxy of high-quality InGaAs and Ge (Sn) plays a pivotal role to boost the research and development of the hybrid SWIR image sensor. Therefore, in this section, we briefly introduce the recent progress in epitaxial InGaAs and Ge (Sn) SWIR FPAs.

### 2.1. Epitaxial InGaAs SWIR FPAs

The mainstream tools to deposit InGaAs are Molecular Beam Epitaxy (MBE) and Metal-Organic Chemical Vapor Deposition (MOCVD). MBE is ideal for the research and development of quantum wells and superlattices (with atomic precision), whereas MOCVD is widely used for the mass production of LEDs, lasers, and photodetectors [59,60,61,62,63,64]. As an example, In_0.53_Ga_0.47_As material is grown on InP wafers as an active layer for SWIR detectors with the spectral response range of 0.9–1.7 μm. Due to the complete lattice matching throughout the entire layer structure, In_0.53_Ga_0.47_As SWIR sensors exhibit extremely low dark current at room temperature [65]. In addition, by tuning the In content of In_x_Ga_1−x_As/InP within 0.53 < x < 1, the cut-off wavelength can be extended from 1.7 μm to the longer wavelengths. This possibility has brought wide attention from both the academic and industrial communities due to their applications in life science, the medical sector, defense, surveillance, security, free-space optics (FSO), thermography, agriculture, food inspection, and LiDAR [49,66,67]. However, in such structures more care is needed due to the lattice mismatch between InP and extended In_x_Ga_1−x_As (0.53 < x < 1), which may increase the dark current by several orders of magnitude [66]. Higher dark current also degrades the signal-to-noise (SNR) ratio and limits the sensitivity and dynamic range of InGaAs SWIR sensors [68,69,70]. To solve the above-mentioned problem, various growth strategies have been investigated, including linearly step, graded buffer layer, compositional overshot, and digital alloy (DA). The progress in InGaAs epitaxial growth enables researchers to precisely control the composition, doping concentration, threading defect densities (TDDs), and thickness of each layer, thereby optimizing the responsivity, dark current, sensitivity, and response speed of InGaAs photodetectors [71,72,73]. Table 1 summarizes the main results of epitaxial InGaAs SWIR FPAs published over the past two decades.

### 2.2. Epitaxial Ge(Sn) SWIR FPAs

In group IV materials, germanium exhibits high compatibility with CMOS process technology, which is cost-effective for Si wafers with a large wafer size [39,80,81] and has an excellent photoresponse in the SWIR band, and an adjustable bandgap (through strain engineering, alloy engineering, and doping engineering). These material properties make germanium an essential candidate for next-generation SWIR sensor technology [82,83,84,85,86]. To enhance the crystal quality of Ge material, various growth strategies have been proposed [87,88,89,90,91,92], e.g., double deposition temperature including low and high temperature, As-doped Ge buffer, ultra-thin SiGe/Si superlattice buffer layer, reversed-graded SiGe buffer, high-temperature H_2_ annealing, cyclic thermal annealing, and selective epitaxial growth (SEG). Thanks to the effort of many researchers in this field, threading defect densities (TDDs) in the germanium epilayers have been significantly reduced to the order of 10^6^ cm^−2^ [93,94,95]. Nevertheless, there are still tremendous mismatch defects located at the interface between the Ge epilayer and Si wafer, with pixels with this configuration featuring high dark current and low peak responsivity at 1310 nm and 1550 nm [96,97,98,99,100]. Consequently, a hybrid Ge/Si SWIR image sensor with the spectral response range of 0.9–1.7 μm cannot compete with the commercially available hybrid InGaAs SWIR image sensor.

To compete with the InGaAs SWIR image sensor, the GOI (Germanium-on-Insulator) structure has emerged as a timely solution [96,101,102]. Numerous methods, such as, smart-cut technique, dielectric wafer bonding technique, and amorphous layer wafer bonding technique, have been adopted to fabricate exceptional GOI wafers with a high-quality top Ge layer [103,104,105]. Thus, the highly defected Ge/Si interface is removed and only the Ge layers with low TDDs are kept, which is beneficial for mitigating the dark currents of GOI pixels [105]. As a result, peak responsivity, peak quantum efficiency, and bandwidth have also been greatly improved due to oxide-induced resonant cavity effects (RCEs), enabling the performance of the GOI SWIR image sensor at the wavelength of 1310 nm to be comparable with or even superior to that of the InGaAs SWIR image sensor product [106]. To address the intrinsic absorption coefficient limitation of Ge at 1550 nm, several reports have presented that a distributed Bragg reflector (DBR) can improve the detection performance and that the process is fully compatible with the GOI process flow [107,108]. Both theoretical and experimental results have confirmed the feasibility of using 2–3 periods of the DBR structure. It can increase the interaction between 1550 nm light waves and the Ge absorption region. As a result, the performance of the GOI SWIR image sensor with a spectral response range of 0.9–1.7 μm is now comparable to that of the InGaAs SWIR image sensor product [109]. Accordingly, the GOI SWIR image sensor with a DBR structure has the greatest potential to replace the InGaAs SWIR image sensor’ market thanks to its CMOS compatibility, cost-effectiveness, and more diverse application scenarios in our daily life (especially in consumer electronics and medical applications) [109].

To extend the spectral response range of the Ge SWIR image sensor beyond its cut-off wavelength of 1.7 μm, the Sn element can be incorporated into the Ge matrix where the direct bandgap emerges for Sn content above 6% [54,110,111]. Experimental results indicate that GeSn image sensors with 1–12% Sn content feature e-SWIR properties, suggesting GeSn image sensors are a promising alternative to e-SWIR InGaAs image sensors in near future [112,113,114]. The development of GeSn e-SWIR image sensors is expected to be rapidly boosted for many applications [115,116,117]. Like the InGaAs e-SWIR image sensor, the GeSn e-SWIR image sensor also faces multiple challenges, including but not limited to the lattice mismatch and thermal mismatch between the GeSn and Si or Ge substrate [118,119,120]. In addition, there are also plentiful challenges that need to be overcome, such as Sn incorporation limitations, Sn segregation, the control of Sn composition, strain stability and TDDs, crystal quality, the improvement of doping concentration, and reliable N-type or P-type ohmic contacts [121,122,123,124,125,126]. Table 2 summarizes the main results of epitaxial Ge (Sn) SWIR FPAs published over the past two decades.

## 3. Flip-Chip Bump Bonding Technology

**Figure 1:**
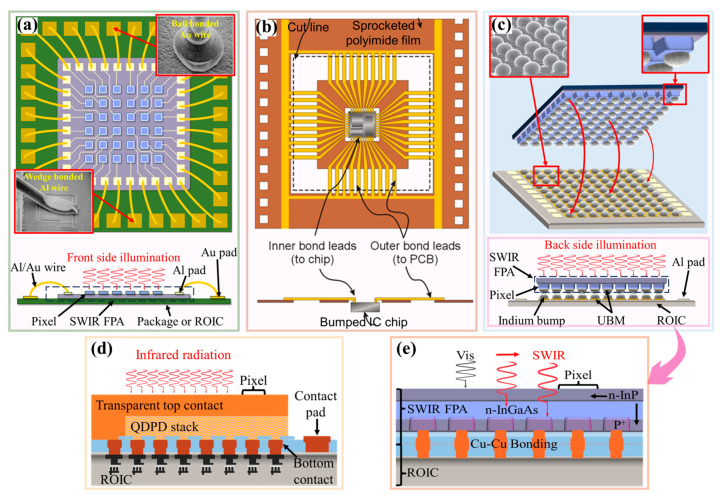
Schematic diagrams of three dominant methods for the electrical interconnections between epitaxial SWIR sensors and CMOS ROICs: (**a**) wire bonding; (**b**) tape automated bonding [140], reprinted with permission from ref. [140], Copyright 2020 SAGE Publications India Pvt; and (**c**) flip-chip bonding. Technology trends for SWIR image sensors: (**d**) monolithically integrated QDPDs on a Si ROIC [20]. (**e**) Cu-Cu hybridized InGaAs SWIR image sensor [57].

SWIR PFAs require a suitable hybridization solution for reliable electrical connections with CMOS ROICs, ensuring package reliability and maximizing their potential performance [135,136,137,138]. Traditional hybrid technologies include wire bonding (WB), tape automated bonding (TAB), and flip-chip bonding [139,140,141,142] (Figure 1a–c). Compared to WB and TAB, flip-chip bonding offers superior electrical performance, high-density interconnections, and improved thermal characteristics, making it ideal for hybrid SWIR image sensors [143]. Flip-chip bonding also employs a face-down structure, allowing the entire surface of an SWIR image sensor and ROIC to be covered with bumps to achieve the highest possible input/output (I/O) count (Figure 1c) [144,145]. Electrical connections are established through soldered bumps at the stacking interface, facilitating uniform power and heat distribution, shorter interconnections, faster signal response, and lower inductance [146]. The flipped SWIR FPA naturally forms a backside-illuminated detection structure, resulting in a fill factor close to 100%. Currently, mainstream flip-chip technology is based on indium bump low-temperature bonding, which is capable of achieving large-scale arrays with 4 K × 4 K pixels [147]. The number of connected indium bump pairs can reach 1.6 × 10^7^, which far exceeds the capabilities of WB and TAB capability. Such a large number of interconnections can be completed with just one flip-align-bonding process.

Solder bumps require sufficient volume to ensure bonding reliability. Therefore, there are challenges in achieving hybrid pixel pitches that are less than 5 μm and high aspect ratios. Moreover, indium-based flip-chip bonding encounters a technological impasse due to a constricted process window, as well as the necessity for enhanced reproducibility, and the restricted scalability of indium bumps. The individualized handling of each die poses a significant obstacle to augmenting the manufacturing throughput of flip-chip bump bonding. This situation asks for urgent attention and the exploration of alternative bonding techniques or process optimizations to overcome these limitations and to ensure the continued progress of relevant technologies. Utilizing CQD layers as absorbers allows QD photodiodes to be fabricated directly on ROICs, and pixel pitch is solely dependent on the bottom contact pads [13] (Figure 1d). Although monolithic array structures based on CQDs have potential to achieve a pixel pitch below 1 μm, they are not reliable for epitaxial image sensors. Sony has developed Cu-Cu bonding with the aim of enhancing productivity and enabling pixel-pitch scaling for back-illuminated InGaAs image sensors [57]. This new process architecture is reported to achieve the same level of dark current density as that of standard hybrid architecture [148]. Cu-Cu bonding, as a formidable contender against indium bump flip-chip bonding, blazes a trail for the development of high-definition epitaxial SWIR image sensors. It offers a promising alternative that holds the potential to revolutionize the field, enabling enhanced performance and greater possibilities in imaging technology. However, further research is still required to investigate the compatibility and reliability of SWIR sensors based on other materials with this process architecture.

Since 2016, Cu-Cu bonding has been developed for the mass production of Si-based stacked CMOS image sensors (CISs), which guarantees high yield and it is expected to reduce the pixel pitch to 1 μm, laying the foundation for scaling down the pixel pitch of InGaAs image sensors [57,58]. Especially, Cu-Cu bonding is promising to enable whole CIS process fabrication for epitaxial Ge (Sn) SWIR sensors. With this comes the need for higher industrial cleanliness to minimize the adverse effects of particles on bonding interfaces. Furthermore, the chip-to-wafer (C2W) process enables small III-V PDAs to be fabricated in large CIS wafer processes with a productive infrastructure and a low level of damage. Using the C2W process in combination with Cu-Cu bonding, a fine-pixel-pitch process architecture with high productivity can be established. Optimized processes using CIS technology and highly precise InP thinning processes facilitate low dark current and high sensitivity to visible as well as SWIR wavelengths. Nevertheless, adopting Cu-Cu bonding cannot avoid the potential reliability problems caused by moisture ingress, electromigration, and copper diffusion, etc [58]. The silicon oxide isolation medium which is used for Cu-Cu bonding is hydrophilic, and the moisture from inside (e.g., materials or substances used in manufacturing) or outside (e.g., water from the dicing step, atmospheric contamination) could lead to the potential risk of moisture sensitivity. Dielectric metal contamination caused by copper diffusion can dramatically deteriorate dielectric reliability, and misalignment or overlay error after bonding can exacerbate copper diffusion along the bonding interface. Additionally, void nucleation and void growth could lead to an electrical resistance increase, and ultimately to an open interconnect. The next challenge for the C2W process is to improve the throughput of III-V chip placement on a wafer for higher productivity. Generally, the effects of different coefficients of thermal expansion (CTE), such as the flatness of the III-V/Si heterogeneous wafer and the bonding process conditions, are still present and become noticeable as the pitch decreases [57].

### 3.1. General Process Flow for Flip-Chip Technology (Part I)

**Figure 2:**
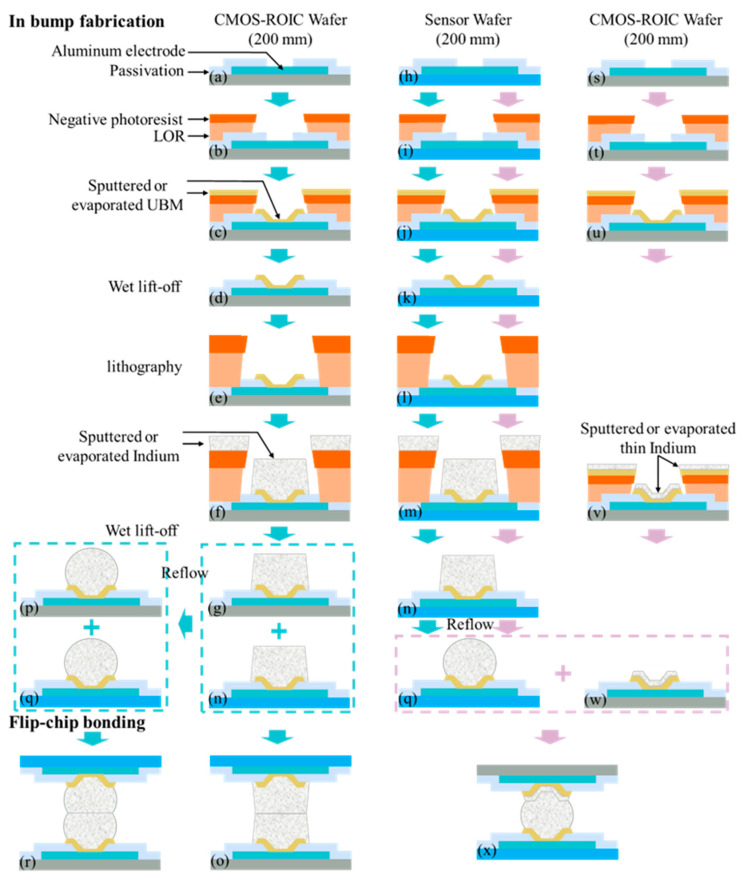
Two conventional flip-chip process flows for epitaxial SWIR image sensors [152]. Dual-side bumping (guided by the blue arrows): (**a**–**g**) indium bump deposition on the CMOS-ROIC wafer; (**h**–**n**) indium bump deposition on the sensor wafer; (**p**–**r**) flip-chip bonding with reflowed and (**g**,**n**,**o**) non-reflowed indium bumps. Single-side bumping (guided by the purple arrows): (**h**–**n**,**q**) indium bump deposition on the sensor wafer with reflow; (**s**–**w**) thin indium deposition on the CMOS-ROIC wafer; (**x**) reflow-assisted self-aligned flip-chip bonding.

Currently, SWIR image sensors employ two types of flip-chip processes: dual-side bumping [149,150,151] and single-side bumping [152,153,154] (Figure 2). The primary distinction between these two processes lies in the arrangement of solder bumps. Dual-side bumping establishes connections on both sides of the chip, whereas single-side bumping focuses on connections on the sensor side [1]. The flip-chip process is introduced with a 200 mm sensor wafer and a CMOS ROIC wafer as then processing targets; ROICs are fabricated using standard Si-based CMOS processes [155,156,157]. Both 8-inch and 12-inch ROIC wafers have reached a mature commercial stage and can be customized according to specific requirements [158,159,160]. Regarding epitaxial SWIR image sensors, there are various types, such as InGaAs and GOI SWIR image sensors. Particularly, concerning the feasibility of the 8-inch GOI wafer process, further optimization and improvement of process stability and reliability are still necessary to ensure optimal performance and high yield rate [89,101,109].

#### 3.1.1. Dual-Side Bumping

Dual-side bumping primarily consists of the following steps: (I) surface passivation of both the CMOS ROIC wafer and SWIR image sensor wafer; (II) exposure of the top electrodes of SWIR pixels and signal reception units (Figure 2a,h) [161,162]; (III) application of under-bump metallization (UBM) [163,164,165] and indium deposition (Figure 2c,f,j,m); (IV) lift-off processes conducted two times to remove excess UBM and indium bumps (Figure 2g,n); (V) reflowing the indium to form spherical indium bumps (Figure 2p,q), or leaving it untreated (Figure 2g,n); and (VI) flip-chip bonding (point to point) with and without indium bump reflow (Figure 2r,o) [152]. Several considerations are essential throughout these process flows.

Firstly, during the exposure process, a hardened thin layer may form at the bottom of the dark regions of the negative photoresist. This is due to the stray light, which cannot be removed after development, and it creates unacceptable isolation between metal and electrodes [166,167]. To avoid this phenomenon, lift-off resist (LOR) can be used. LOR is not light-sensitive and adding it between a negative photoresist and substrate can prevent the formation of the hardened layer. Furthermore, LOR facilitates the creation of the desired “undercut” (Figure 2b,i) [168]. For thicker indium (≥5 μm) lift-offs, a spin-coated negative photoresist may not provide sufficient thickness. In such cases, placing LOR beneath the negative photoresist can increase the overall photoresist thickness (Figure 2f) [169]. It is important to note that LOR requires specific solvents for removal [170]. Secondly, when a thicker photoresist is not feasible, the dimensions of the indium bump pattern can be appropriately enlarged to form an “indium disc”, ensuring sufficient indium volume [171]. The height of the reflowed indium bump mainly depends on the deposited indium volume. If reflow is not employed, a thicker photoresist and high-quality indium deposition are required. The height of the indium bump after lift-off represents the target height (Figure 2g,n). The reflow should be performed to improve the morphology of the indium bump if it is suboptimal post-lift-off (Figure 2p,q). Thirdly, dual-side bumping is well suited for fine-pitch (≤10 μm) cold compression bonding, which can perform at room temperature or below the melting point of indium. This method is capable of accommodating a certain degree of warpage and flatness variations in large-scale (4 M pixels) SWIR FPAs [42,56,172,173]. In addition, reflow is a not suitable process for dual-side bumping once flip-chip bonding has been performed.

#### 3.1.2. Single-Side Bumping

Single-side bumping refers to the process of bumping on the sensor wafer (Figure 2h–n), while the depositing UBM occurs on the CMOS ROIC wafer (Figure 2s–u). To enhance the adhesion and bonding strength, a thin indium layer is deposited prior to the UBM deposition of the CMOS ROIC wafer (Figure 2v). Following this, both the UBM and indium are lifted off (Figure 2w), so one lithography step is eliminated. The photoresist thickness obtained in Figure 2t must exceed the one that is in Figure 2b,i to prevent adhesion during indium deposition (Figure 2v). Figure 2x illustrates point-to-point flip-chip bonding, where reflow contributes to self-alignment (Figure 2q) and enhances the mechanical strength [174,175]. During reflow, the chip with indium bumps should be positioned underneath. For smaller-scale SWIR FPAs with larger pitch and less precise flip-chip bonding requirements, single-side bumping offers cost advantages. Regardless of whether single-side or dual-side bumping is employed, both the SWIR image sensor wafer and CMOS ROIC wafer must be diced into individual chips prior to flip-chip bonding. This is due to the fact that current flip-chip bonding machines mostly guarantee the yield and feasibility of either chip-to-chip bonding or chip-to-wafer bonding [176,177].

### 3.2. General Process Flow for Flip-Chip Technology (Part II)

**Figure 3:**
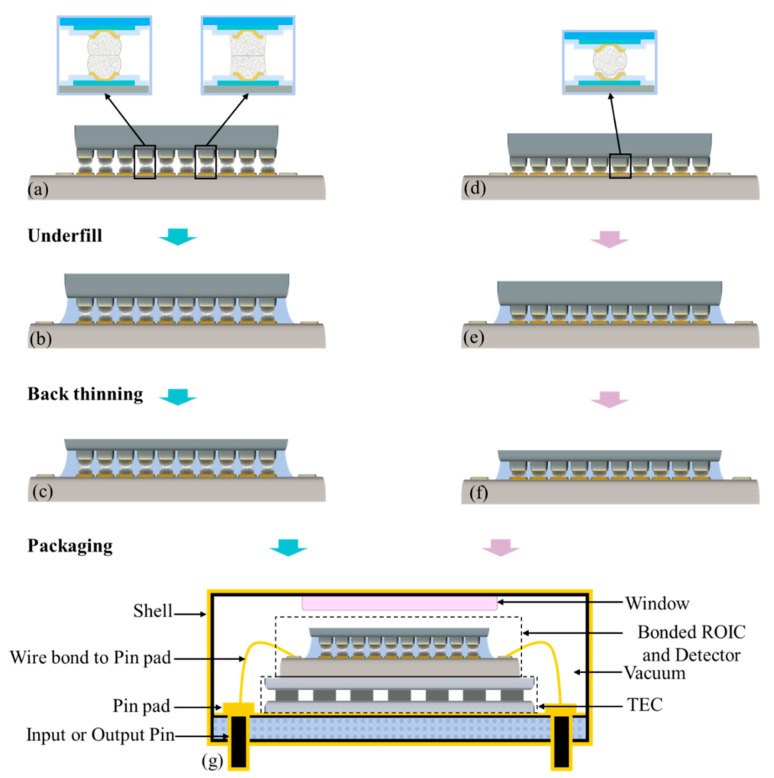
The second part of the conventional flip-chip process. Underfill, back thinning, and packaging for flip-chip-bonded SWIR image sensor with (**a**–**c**) dual-side bumping; (**d**–**f**) single-side bumping; (**g**) packaged SWIR image sensor for room-temperature operation. The enlarged regions in (**a**,**d**) illustrate the point-to-point indium connection with different bumping conditions.

Part II is dedicated to improving the stability, reliability, and functionality of SWIR FPAs through processes such as underfill and back thinning, ultimately aiming to provide true usability through packaging. Figure 3a,d illustrate the structures of SWIR FPAs after flip-chip bonding, where the sole mechanical connection between the SWIR image sensor and CMOS ROIC is established through indium bumps. Once cured, the underfill material forms a mesh-like structure that encapsulates the indium bumps, isolating them from air and mitigating thermal stress across the entire SWIR FPA bare module (Figure 3b,e). Achieving a near-100% filling rate with underfill close to 100% is essential to prevent the noise points in imaging that may arise from unfilled tiny gaps.

Epitaxial SWIR image sensors require back thinning and polishing treatment of their backside (Figure 3c,f). In the areas where indium bumps are absent, underfill plays an important supporting role to relieve the local collapse caused by back thinning [178]. Applying an anti-reflection coating to the thinned surface can notably reduce the incident radiation loss (not depicted in Figure 3). The packaging form varies depending on the application requirements. A commonly used room-temperature metal shell package flow is briefly introduced in Figure 3g. This metal shell serves as an effective shield against external electromagnetic interference and features both inner and outer surfaces that are gold-plated. The upper cover of the package includes an incidence window made of sapphire or quartz [179,180]. The I/O pins located at one end inside the package are gold-plated pads that are wire-bonded to the ROIC. At the other end outside the package, there is an electrical interface that connects to the signal acquisition and processing system [181,182].

The package is commonly paired with a compact thermoelectric cooler (TEC) [183,184,185]. The backside of the SWIR FPA is aligned with the window to guarantee that the entire incident surface can receive infrared radiation [186]. The upper cover and base of the package are sealed and welded in a dry inert gas environment (or vacuum), with gold coating enhancing weld strength and ensuring effective sealing. The metal shell package creates a protective environment for the SWIR FPAs, safeguarding them from physical damage and external contamination. Additionally, it provides optical, electrical, and mechanical interfaces, thereby enhancing the system’s reliability and environmental adaptability [187,188,189].

### 3.3. Indium Bump Formation

**Figure 4:**
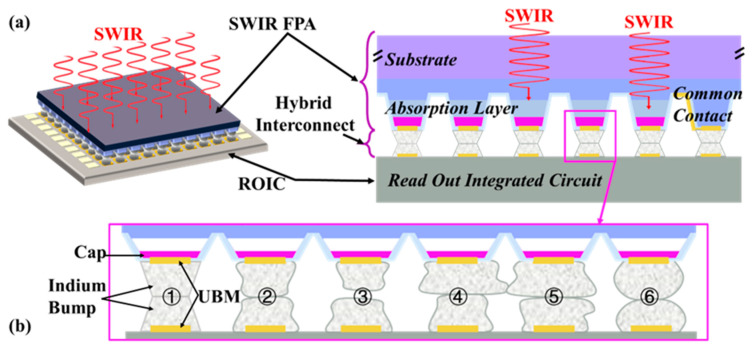
(**a**) Schematic diagram of the structure of flip-chip hybridized SWIR FPAs. (**b**) Indium bump under different conditions: ① ideal indium bump connection; ② common-shape indium bump in actual manufacturing; ③ shorter indium bump leads to open-circuit connection; ④ and ⑤ short-circuit indium bump connection; ⑥ reflowed indium bump connection.

**Figure 5:**
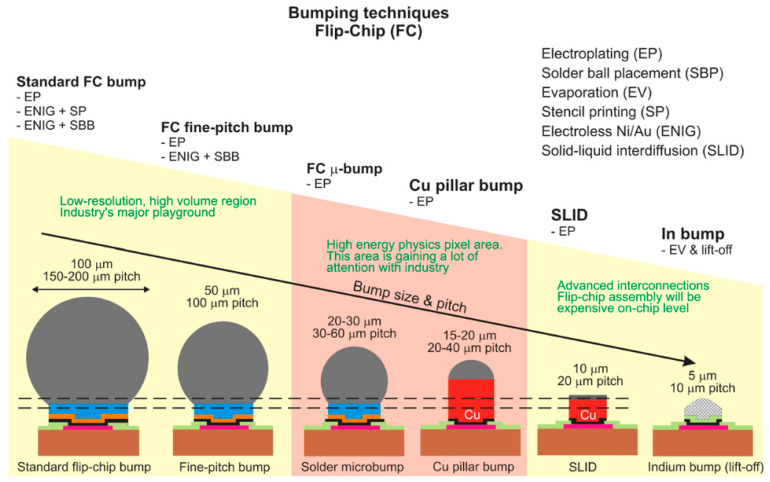
Various bumping technologies used in the manufacturing of epitaxial image sensors [11]. Reprinted with permission from ref. [11]. Copyright 2022 IOP Publishing.

SWIR FPAs have reached a high level of maturity in imaging applications, and the hybridizing integration of SWIR FPAs and CMOS ROICs through micro-connections and flip-chip bonding is widely acknowledged as a viable technological approach [177,186,190,191] (Figure 4a). To accommodate flip-chip bonding, independent metal micro-bumps are employed instead of metal wires, enabling high-density interconnections. Figure 5 depicts the various bumping technologies employed in flip-chip bonding and their process evolution [11]. Common bumping materials include Sn [192,193], Sn-Pb [194,195], Au-Sn [196,197], Sn-Ag [198,199], Cu-Sn [200,201], In [149,152,177,202], etc., which are characterized by their soft texture and robust bonding strength [165]. The characteristics and yield of micro-bump arrays directly influence the imaging quality [203,204,205]. The careful selection of bumping materials and optimization of associated processes significantly enhance the reliability and advancement of hybridized SWIR imager sensors.

Indium is highly esteemed in the SWIR imaging field due to its exceptional physical and chemical properties [206,207]. Its remarkable low-temperature operability (such as liquid nitrogen at 77 K or even liquid helium at 4.15 K [140,206]) has made the indium bump the preferred choice, particularly in military applications [140]. Pure indium offers several advantageous properties, including a low melting point (156.6 °C), low yield stress, high thermal conductivity, and excellent wettability [208,209,210]. Indium exhibits excellent ductility and plasticity across a wide temperature range, making it ideal for assembling and connecting heat-sensitive or mechanically delicate devices [211,212]. Additionally, indium effectively mitigates the stress caused by a thermal mismatch [213] and remains the optimal material choice for applications involving thermal cycling [214].

The low melting point of indium facilitates its bonding capability at room temperature, thereby preventing undue stress on connections and reducing the thermal budget of related processes [202,212]. Moreover, the high plasticity of indium ensures that the SWIR FPA and CMOS ROIC are less susceptible to mechanical damage during assembly. The deformation that occurs during flip-chip bonding alleviates stress between the SWIR FPA and CMOS ROIC, and it accommodates warpage [152,202]. SWIR imaging requires solder bumps with micrometer-scale dimensions, and indium can perfectly fulfill these demands. Typical indium bumps range from 5 to 30 μm in diameter and offer excellent electrical characteristics, such as acceptable resistivity and low inductance, which are crucial for high-frequency operation [215,216]. The dense stacking of indium bumps significantly improves the device’s signal-to-noise ratios [217,218]. Whether for cooled or uncooled SWIR image sensors, indium proves highly compatible as an electrical interconnect material. The fabrication process of indium bump consists of two main steps: (I) under-bump metallization; (II) indium bump formation.

#### 3.3.1. Under-Bump Metallization

UBM consists of multiple layers, and each metal layer has a specific function. Aluminum (Al) serves as a common bonding pad material for both CMOS ROICs and SWIR FPAs. However, the formation of aluminum oxide (Al_2_O_3_) film hinders the wetting of indium on aluminum, making it necessary for the UBM layer to act as an intermediary between the indium and Al pads [219]. A commonly used UBM structure includes Titanium (Ti, as an adhesion layer), platinum (Pt, as an indium diffusion barrier layer), and gold (Au, as an indium wettable layer) [163,165]. Additionally, the UBM configuration also influences the formation of ohmic contacts. Ti, known for its strong adhesion properties, is employed as the bottom layer of the UBM, forming a durable bond with Al. The middle layer typically consists of a barrier metal (e.g., Ni and Pt), creating a Ti/Pt or Ti/Ni structure that effectively prevents the downward diffusion of indium [220]. At the topmost layer, Au is utilized due to its excellent wetting characteristics with indium, securely anchoring the indium bump onto the UBM. The reaction between Au and indium forms a continuous interface layer of a AuIn_2_ intermetallic compound (IMC), which enhances resistance against indium diffusion [221,222]. During high-temperature processes like reflow, Au continues to react with indium and makes a sufficient thickness to prevent depletion [212,223,224]. Although both Au and indium are soft, ductile metals, AuIn_2_ possesses a harder texture that can impact the overall reliability of UBM and indium bumps [225,226]. UBM deposition can be achieved through conventional methods, such as evaporation [202,227,228,229,230], sputtering [152,231,232], or physical vapor deposition [233]. Table 3 outlines UBM options which are suitable for an indium bump system.

**Figure 6:**
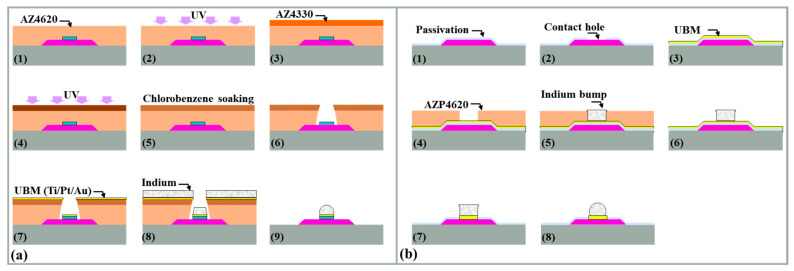
Process flow for indium bumping. (**a**) Evaporation: (**1**) AZ4620 photoresist coating; (**2**) flood exposure for AZ4620; (**3**) AZ4330 photoresist coating; (**4**) exposure for AZ4330; (**5**) chlorobenzene soaking; (**6**) photoresist development; (**7**) UBM deposition; (**8**) indium deposition; (**9**) lift-off and indium bump reflow. (**b**) Electroplating: (**1**) passivation deposition; (**2**) contact hole etching; (**3**) UBM (seed layer) deposition; (**4**) AZP4620 photoresist patterning; (**5**) indium electrodeposition; (**6**) photoresist removal; (**7**) UBM etching; (**8**) indium bump reflow.

The role of UBM varies depending on the method used to manufacture indium bumps [202] (Figure 6 shows details on indium bump formation via evaporation, and electroplating follows). Figure 6a demonstrates the image reversal process using a positive photoresist, where both the UBM and indium bumps are achieved in a single-step wet lift-off. In contrast, Figure 6b shows a process where an additional passivation layer is required before UBM deposition, and bumps are formed through electroplating, followed by photoresist removal. Subsequently, most of the UBM is removed via dry etching. Herein, UBM serves its original function and also acts as a seed layer for electroplating. It is important to note that dry etching inevitably results in some of the loss of indium bumps; the passivation layer only protects the underlying structure from forming the damage. Alternatively, a patterned UBM (seed layer) can be obtained through wet lift-off instead of etching. Both methods involve indium bump reflow, during which the molten indium forms a truncated spherical shape due to surface tension. Circular UBM is commonly used to facilitate the sphericalization of indium bumps. During reflow, the indium bump aligns automatically with UBM and remains within the UBM region; hence, UBM is also referred to as ball-limited metal (BLM) [224].

#### 3.3.2. Indium Bump Formation

The significance of indium in flip-chip bonding stems from its capacity to establish reliable electrical and thermal connections while meeting the mechanical tolerances and thermal management requirements of contemporary semiconductor devices. Its softness, low melting point, conductivity properties, and compatibility play a crucial role in enhancing the efficiency, performance, and durability of flip-chip assemblies in image sensor manufacturing. Typically, the process of forming indium bumps involves designing the bumps, depositing indium, and optimizing their characteristics.

##### Indium Bump Design

The pitch and quantity of indium bumps are determined by the pixel design of SWIR FPAs. Typically, SWIR FPAs have a pixel pitch of 10 μm or even larger, although there are exceptions with pitches as small as 7–5 μm. Indium bumps serve as intermediaries between the electrical and mechanical connections of each pixel and signal processing unit, establishing point-to-point contact. CMOS ROICs used in SWIR FPAs imaging are generally commercial and characterized by standardization, maturity, and systematization. As a result, the design and layout of SWIR FPAs are tailored to fit CMOS ROICs, which reduces design costs and streamlines subsequent process. Once the design of SWIR FPAs is finalized, the number and spacing of indium bumps are determined accordingly. Several factors must be considered in determining the shape and size of the cross-section of indium columns [234]: (I) the strength of flip-chip bonding depends on the contact area between indium bumps, and larger contact areas can withstand higher bonding pressure, resulting in stronger overall bonding [235]; (II) a larger cross-sectional area implies lower resistance in the indium bumps; (III) the diameter or width of indium bumps should closely match the pixel size while adequately covering the UBM.

Indium bumps typically assume a cylindrical form due to the opening effect of the photoresist [217]. Figure 4b(①) illustrates the longitudinal cross-section of ideal indium bumps, while Figure 4b(②) shows the common shape observed in actual manufacturing. As the number of pixels increases and pitch decreases, indium bump reflow shaping is used to enhance the success rate of hybrid bonding (Figure 4b(⑥)). Pressure is applied in the flip-chip bonding process. This pressure is transmitted to and exerted on the indium bumps, leading to a controlled collapse at contact points. The accumulation and lateral expansion of indium bumps at contact points result in the horizontal section diameter being greater than originally designed [143]. The size and pitch of the pixel needs to be considered in the design to prevent short circuits, particularly at fine pitches (Figure 4b(④,⑤)). Under conditions of acceptable bonding strength, this variation can be effectively controlled by the precise management of welding pressure.

The height design is flexible within a certain range, which provides the result of the height difference between indium bumps remaining acceptable. Within the same height range, shorter indium bumps may lead to weak solder joints after flip-chip bonding, whereas higher indium bumps are prone to adhesion between adjacent indium bumps (Figure 4b(③,④)). Therefore, ensuring uniformity in height within the same batch is crucial. The overall height of indium bumps should not be too low, and increasing the height of indium bumps offers several advantages: (I) there is enhanced tolerance for warping in SWIR FPAs and CMOS ROICs [230]; (II) higher indium bumps provide more plastic deformation to absorb mechanical stress [202]; and (III) an increased bonding gap after flip-chip bonding facilitates the capillary flow of epoxy resin, ensuring complete underfill to enhance the bonding strength [236]. The reference aspect ratio of indium bumps is limited to not less than 1:1, which is believed to help reduce coupling noise between pixels [214].

The surfaces of SWIR FPAs and CMOS ROICs are not perfectly flat. Therefore, it is necessary to take the surface morphology into account when deciding whether to increase the margin for indium deposition. The surface structures of planar-type SWIR FPAs and CMOS ROICs are similar, with each of them featuring a passivation layer above the pixel electrodes or signal processing units, which include contact windows. The passivation thickness should not be underestimated, especially when it approaches the pitch size. Contact windows in the passivation have a specific volume that consumes a portion of indium. In mesa-type SWIR FPAs, isolation trenches are etched between pixels to reduce electrical crosstalk. Consequently, the surface structure and trench isolation flatness are totally different from those of the planar-type SWIR FPAs. Although isolation trenches increase the challenge for achieving well-patterned lithography, they prove advantageous for the speed and success rate of underfill. While UBM enhances the surface flatness to some extent, the metal thickness typically remains thinner than 500 nm, and this limits its effectiveness in achieving perfect flatness.

**Figure 7:**
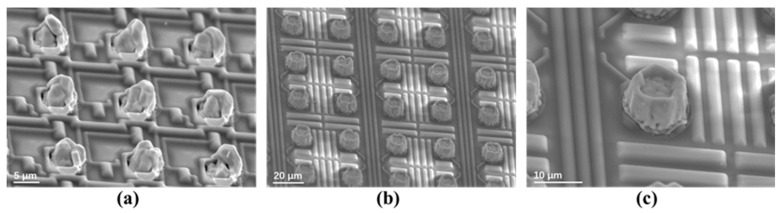
(**a**) Insufficient and (**b**) sufficient deposition of indium bump on ROIC (after lift-off); (**c**) pit-like collapse on the upper surface of indium bump.

Figure 7 illustrates both insufficient and sufficient indium deposition conditions on the CMOS ROIC. In Figure 7a, it is evident that insufficient indium results in a significant portion being consumed by the passivation window. Indium bumps under these conditions fail to meet the flip-chip bonding standards, particularly at the wafer’s edges where insufficient indium is often observed. Thus, improving the deposition processes or upgrading equipment could potentially mitigate this issue. In Figure 7b, indium bumps on the CMOS ROIC exhibit sufficient deposition where the passivation of windows is fully achieved and well filled, and the indium bump height is adequate for subsequent processes. Figure 7c highlights the pit-like features on the upper surface of indium bumps, emphasizing the impact of indium consumption by passivation windows.

Different flip-chip bonding methods necessitate distinct approaches to fabricating indium bumps. In the single-side bumping process as discussed earlier, one-side indium bumps’ quality strongly determines the yield of flip-chip interconnects. The reflow of indium bumps is indispensable to achieve uniform heights. In contrast, the dual-side bumping process allows for higher tolerance. Cold compression flip-chip bonding can provide high pixel operability without indium bump reflow. This is mainly due to the high aspect ratio of dual-side indium bumps after cold compression bonding at room temperature (Figure 4b(①)), effectively reducing the stresses induced by thermal mismatch between SWIR FPAs and CMOS ROICs [143]. Moreover, it compensates for the lateral alignment shifts which are caused by chip warpage. Consequently, the dual-side bumping process offers advantages, especially for very-large-format SWIR FPAs.

In scenarios where reflow is necessary (indium discs are transformed into indium bumps [152]), special attention should be paid to the UBM dimension and lithographic pattern of indium, as well as the indium thickness. Under ideal conditions, the indium bump height after reflow can be determined using the volume formula for truncated spheres [237]:

$$V=\pi (\frac{h^{3}}{6}+h\frac{r^{2}}{2})$$

where *V*, *h*, *r* are the volume, height, and truncated surface radius of the truncated sphere, respectively. *V* is equal to the volume of deposited indium, which can be obtained by multiplying the area of the indium pattern by the thickness of deposited indium (assuming the total volume of indium remains constant before and after reflow). *h* and *r* correspond to the height of the indium bump and the radius of UBM, respectively. In practical situations, the final shape of the indium bump is influenced by the gas atmosphere, temperature, and humidity of the reflow environment [203]. The indium bump may not strictly adhere to the shape of a truncated sphere, and the actual radius may be slightly smaller than *r*, and this is related to the density of indium before and after reflow. When the diameter of the indium bump pattern is less than 10 μm, the opening diameter of the photoresist is generally smaller [217]. When the thickness of indium deposition is fixed, if the horizontal size of the indium pattern is much bigger than the UBM diameter, it will result in an overall excessive volume of indium, making it unable to be fully collected by the UBM and leaving residual indium around the UBM. Conversely, if the lateral dimension of the indium lithography pattern is too small, there will not be enough indium to form an indium bump. In the case of controlling the thickness of deposited indium, Lee et al. tested the different patterning size combinations between UBM and indium bump. The study shows that when the horizontal size ratio between UBM and indium pattern is 1:2.5, UBM could exert the wetting effect to the greatest extent, which reduces the surrounding residual indium [238].

##### Indium Bump Deposition

Electroplating (also referred to as UV-LIGA) and thermal evaporation are two widely adopted methods for indium deposition, due to their low production cost and operational simplicity. Electroplating offers precise control over the deposition rate but typically results in lower quality indium films. It is favored for manufacturing indium bump arrays with larger pitches and diameters [239]. On the contrary, thermal evaporation provides superior control over film uniformity, especially on large wafers (≥8 inches) [217] compared to that of electroplating. However, when dealing with the fine pitch (≤10 μm), which implies a high aspect ratio and large scale, it also poses challenges for achieving high uniformity and low-defect indium bumps with low defect density.

##### Indium Electrodeposition

Electroplating is a straightforward and cost-effective method, producing indium bumps with a fuller morphology, especially when dealing with high aspect ratios [240,241,242]. Key factors influencing electroplating include current distribution and mass transport [243,244]. The uniformity of electroplated indium film is influenced by pattern design. When electroplating indium bump arrays, the planar dimensions (diameter or width) of the bumps are unchanged, while their height can vary depending on the bump density across the wafer. To maintain uniformity, “dummy” or “filler” bumps are often employed at the chip edges [230]. The effective removal of bubbles between the plating solution and the substrate is achieved through stirring to the pre-wet substrate [242]. Height discrepancies in bumps arise from variations in the current density between the central and edge regions of the wafer, where the edge region connected to the cathode experiences higher current density than that of the central region. This effect is more pronounced on high-resistance substrates [240]. Several reports have demonstrated that combining the ultrasonic agitation of plating solution with pulse plating techniques can promote indium bump morphology [240,241].

**Figure 8:**
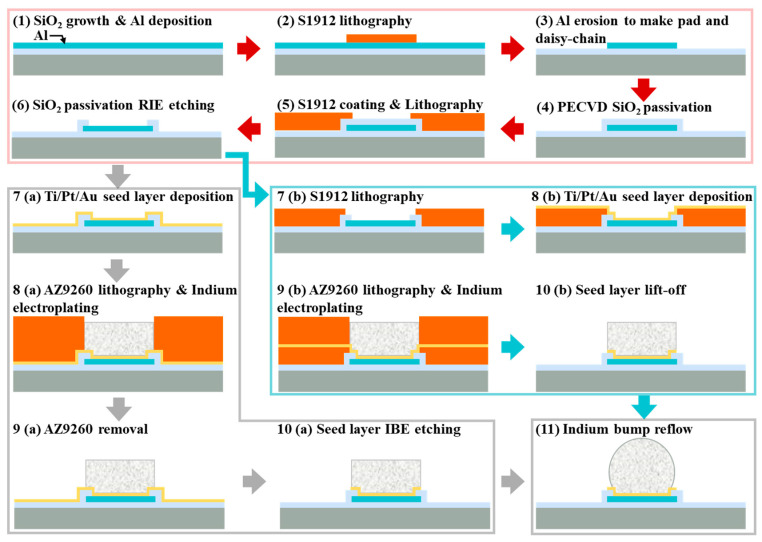
Two schematic flows of the electroplated indium bumping process. (**1**–**6**) Al pad formation on Si wafer. Etching-based ((**7**–**10**) (**a**)) and lift-off-based ((**7**–**10**) (**b**)) removal of the seed layer. (**11**) Indium bump reflow.

A conductive seed layer needs to be formed on the substrate surface before lithography in the electroplating process [202,212,240]. The undesired portions of the seed layer can be removed through etching or lift-off techniques while preserving the indium bumps and pre-existing device structures on the substrate [244]. However, it has been demonstrated that it is unfeasible to avoid damage to indium bumps during Ti/Pt/Au seed layer etching due to the high reactivity and fragility of indium. Huang et al. elaborated on indium bump electroplating, focusing on etching-based and lift-off-based removal of the seed layer, as shown in Figure 8 [212]. The effectiveness of the lift-off process in removing the stubborn Ti/Pt/Au seed layer was verified. During the Ion Beam Etching (IBE) process, Ti/Pt/Au particles would adhere to the indium bump surface, and the temperature of the surface was higher than the melting point, which increased particle adhesion. The contaminated surface would disrupt the surface tension of the molten indium during the reflow process, resulting in reflow failure. In contrast, after the lift-off-based seed layer removal, the indium bump morphology was significantly improved.

**Figure 9:**
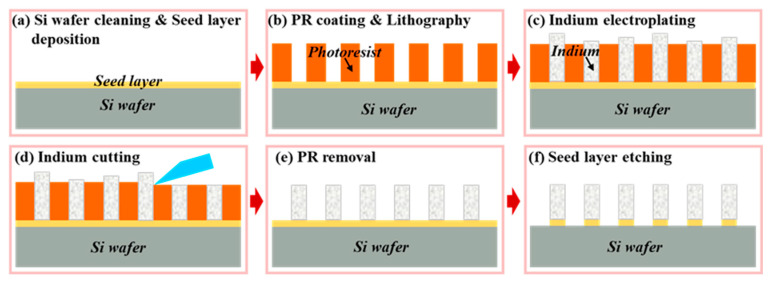
Schematic of electroplating bumping process with indium cutting.

Son et al. successfully developed uniform and high-aspect-ratio (≥2:1) indium bump arrays on 8-inch silicon wafers using electroplating. The group used an indium bump cutting process, and a minimum pitch of 5 μm could be obtained [228]. Figure 9 illustrates the electroplating process with a Ti/Au seed layer. In this process, before electroplating, the wafer undergoes pre-wetting using a stirring method to eliminate bubbles between fine-pitch patterns, using an aminosulfonic acid salt solution as the plating solution. Then, after electroplating, indium bumps are cut and leveled parallel to the wafer surface without removing the photoresist. This method serves as a benchmark for achieving ultrafine pitches in large-format and high-resolution hybrid SWIR FPAs. However, for precision cutting equipment, specific types of photoresists are necessary for the cutting and leveling processes.

##### Indium Evaporation

Thermal evaporation enables rapid film formation with low cost, which is advantageous for depositing micrometer-thick indium films [143,152,245]. Source material (indium) is placed in a crucible and heated until the indium melts. The molten indium is further heated to form an evaporation flow that radiates upward and encounters the patterned substrates, condensing on the surface to form a film. Due to the directivity of evaporation, the step coverage capability of evaporatively deposited indium films is limited, which in turn favors lift-off. The indium bump morphology is determined by several major factors during evaporation, including substrate conditions, photoresist lithography, vacuum level, deposition rate, etc. [143]. The target substrate is fixed on a water-cooled base, which enables heat exchange with the entire system [152]. Rapid and effective heat exchange ensures a constant low temperature of the target substrate and is beneficial for achieving smaller grain size indium. At low temperature, the diffusive mobility of indium atoms is reduced, slowing down the lateral growth of the film and promoting the formation of a flat upper surface of indium bumps [143]. Moreover, the volatilization of residual solvents in the photoresist is suppressed, decreasing the impact on cavity vacuum, and the thermal budget of photoresist is reduced, avoiding overheating to increase the difficulty of lift-off. In this process, the film surface roughness due to island growth decreases with increasing substrate temperature [217].

**Figure 10:**
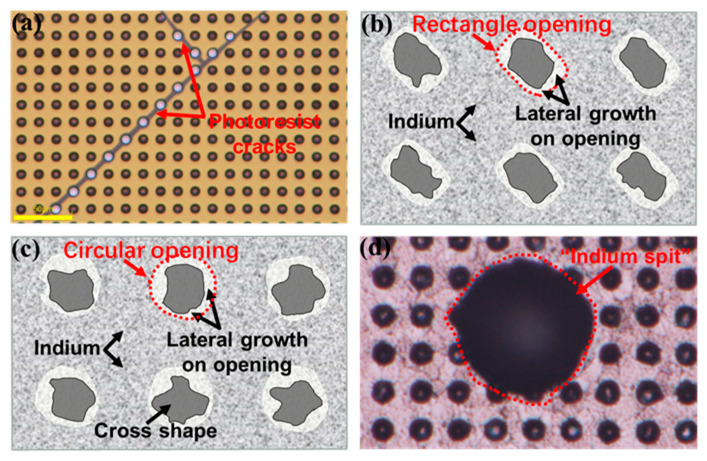
(**a**) Photoresist cracking; (**b**) rectangle photolithography openings before lift-off; (**c**) circular photolithography openings before lift-off; (**d**) “indium spits”.

Photoresists play a critical role in the indium deposition. First of all, the low-temperature stability of the photoresist should be ensured to prevent indium from leaking to the target substrate surface after photoresist cracking (Figure 10a). The shape and roughness of the photoresist opening also significantly influence the indium bump morphology. In order to deposit indium bumps with a large aspect ratio, the top opening size should be increased as much as possible under the premise of a sharp and clear undercut, and a smooth photoresist surface. Higher roughness at the opening implies more nucleation sites and accelerated the lateral growth of indium. The vertical deposition rate of indium is equal to the evaporation rate, but the lateral deposition rate is not directly controlled. For a larger perimeter-to-area ratio (e.g., circular) opening, there is a higher possibility for being completely covered before reaching the desired indium height. Figure 10b,c illustrate the impact of lateral growth on photolithography on openings before lift-off.

The evaporation occurs within a high vacuum to minimize the interactions of vaporized indium atoms with other gas molecules, thereby reducing contamination from gas-phase nucleation. The low-pressure conditions increase the average free path of indium atoms, resulting in an improved indium bump morphology. Excessive deposition rates elevate the partial pressure of indium atoms in the chamber, promoting gas-phase nucleation and cluster formation. Large indium particles escaping can cause “indium spits” (Figure 10d), leading to un-uniform film [217]. The indium evaporation conditions need to be tailored to meet specific application requirements. Precise control and optimization of the deposition rate and chamber vacuum level are fundamental for achieving high-quality indium bumps [143]. For indium bumps with a low aspect ratio (≤1:1), a moderate evaporation rate and optimized substrate temperature are recommended. Cryogenic substrate temperatures are conducive to achieving high aspect ratios (≈2:1). However, it is essential to consider the nonlinear relationship between the photoresist opening closure rate and evaporation rate, as well as the competing effects of the surface migration of indium atoms and the evaporation rate [217].

##### Lift-Off Process vs. Ion Etching

**Figure 11:**
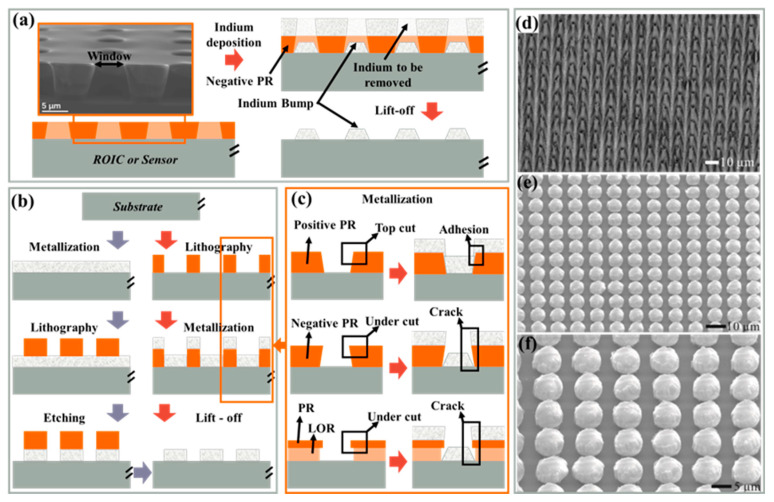
(**a**) Indium bump lift-off process. Inset (**a**) shows the cross-sectional SEM image of the patterned negative photoresist; (**b**) comparison of metal lift-off process (guided by the deep red arrows) and metal etching process (guided by the purple arrows); (**c**) photoresist morphology corresponding to positive and negative photoresists in UV lithography; (**d**) SEM photograph of an array of indium bumps after ion etching, reprinted with permission from ref. [247], Copyright 2020 SPIE; and (**e**,**f**) SEM top-view images of indium bumps after reflow [247], reprinted with permission from ref. [247]. Copyright 2020 SPIE.

The indium residual outside the indium bump array is removed through lift-off [246], as shown in Figure 11a. Indium bump patterns are transferred to the negative photoresist via lithography, appearing as open windows, and then indium is deposited globally. The negative photoresist serves as a mask to prevent indium deposition in unwanted regions. Lift-off solution dissolves the photoresist through the cracks and removes the indium on the photoresist, thereby achieving the patterning of indium.

Lift-off and metal etching (wet etching and dry etching) are common processes for metal patterning, as depicted in Figure 11b. For the lift-off, self-patterning occurs simultaneously with metallization, eliminating the subsequent etching. Indium bumps are higher than conventional metal electrodes and interconnects [248]; then, the etching is less selective and carries a higher risk of damaging the substrate. For indium bump arrays with a pitch exceeding 10 μm, the lift-off process is often more practical and easier to implement. Positive and negative photoresists exhibit “topcut” and “undercut” after lithography, respectively, as shown in Figure 11c. Undercut aids in separating the indium from the photoresist at step edges, while topcut can result in continuous indium film wrapping around the step edges, making it difficult for the lift-off solution to contact the photoresist. In addition to using a negative photoresist to achieve undercut sidewalls, image inversion and a dual-layer photoresist can also be employed [249]. Lift-off is widely recognized as a main cause of non-uniformity, where issues such as inadequate height and adhesion between indium bumps are more pronounced as well [213]. Zhang et al. replaced the lift-off with ion etching to precisely etch indium bumps and UBM [247]. The etched indium bumps exhibited high uniformity, with a 10 μm pitch and an approximately 9.3 μm height (2% uniformity), indicating minimal indium amount variation among the bumps. At this stage, the indium bumps appeared conical and did not meet the requirement of a flat top with a larger area, as illustrated in Figure 11d. Adjustments were made using reflow; the average heights measured were 7.42 μm (Figure 11e) and 7.34 μm (Figure 11f). However, the challenges associated with this method should not be underestimated. Critical considerations include the role of the photoresist in substrate protection, the necessary etching ratio, and the impact of impurities produced during etching on the reflow, which have to be given attention (Figure 11f). Combining etching and reflow can yield ultrafine pitch indium bump arrays with satisfactory height and uniformity, providing practical insights for ultrafine-pitch hybrid applications.

To minimize the adverse effects of lift-off on indium bump arrays and enhance yield rates, several considerations can be taken into account: (I) The photoresist thickness must be adequate to induce cracking at step edges without compromising lithographic resolution. Spray-coating typically results in a thicker photoresist compared to that of spin-coating using the same material, and multiple rounds of spin-coating can also be considered as an alternative to achieve the desired thickness. (II) Temperature control from the lithography stage to the end of the lift-off process should be carefully managed. The temperature during photoresist baking and throughout the deposition process should not exceed safe limits to prevent adverse effects. (III) Ultrasonic treatment or heating can be considered to accelerate the lift-off process. However, prior to this, any already detached indium film should be removed to prevent unintentional scraping of the indium bumps, which could damage the array.

##### Indium Bump Optimization

Before flip-chip bonding, indium bumps typically undergo pretreatment to eliminate indium oxide and impurities using physical or chemical methods. Due to limitations in fabrication, indium bumps produced through evaporation and lift-off may exhibit irregular morphology, including uneven heights and closely spaced neighboring bumps due to top tilting, etc. [203,217]. Similar issues can arise with electrodeposition, underscoring the importance of indium bump optimization before flip-chip bonding. This process is commonly conducted in an oxygen-free environment or with isolation media like glycerol [224,225]. Reflow is often employed to reshape the indium bumps into a spherical shape and appropriate anti-oxidation measures (e.g., photoresist coating) are maintained until the flip-chip bonding begins.

Indium oxide increases the series resistance of indium bumps and diminishes bonding strength [250,251,252]. Therefore, it is essential to prevent oxidation or remove indium oxide before flip-chip bonding. Indium bumps oxidize rapidly upon exposure to air and grow faster in humid air, with their thickness increasing over time [252]. The oxidation rate is highest on freshly cleaned surfaces and correlates closely with relative humidity, likely due to how water vapor condensing on the surface accelerates oxidation [203]. Depositing a layer of gold or silver immediately after indium deposition can serve as an effective anti-oxidation barrier, while this approach is typically suited for non-reflowed indium bumps [215]. A fresh photoresist coating can also protect delicate indium bumps from physical damage, prevent oxidization, and prolong the shelf life [42]. Effective methods to remove indium oxide include plasma etching, acid baths, wet chemical reduction, and using formic acid (HCl, H_2_, CH_4_, and other reducing gases, as well as a hydrogen radical strong reducing agent) in a N_2_ (He, Ar) atmosphere [250,253,254,255,256,257].

**Figure 12:**
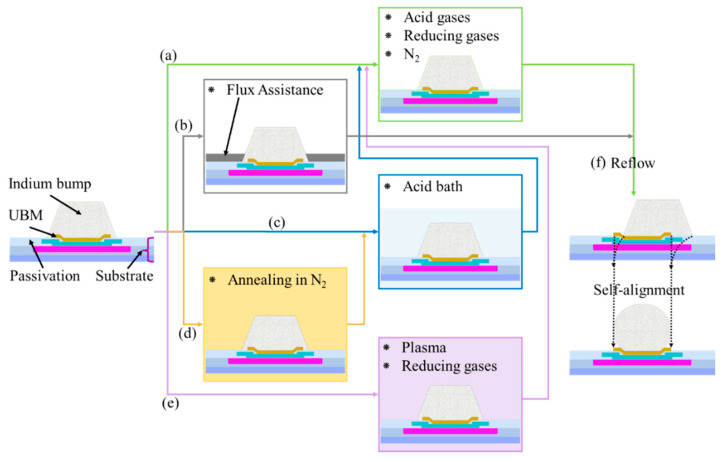
Common optimization methods for indium bumps. (**a**) Reflow in the atmosphere of acidic or reducing gases (HCOOH, HCL, H_2_, CH_4_) (N_2_ as carrier gas); (**b**) flux-assisted reflow; (**c**) acid bath pretreatment prior to the operation of method (**a**); (**d**) annealing in N_2_ atmosphere prior to the operation of method (**c**); (**e**) plasma etching to remove the oxide layer prior to (**a**); (**f**) self-alignment of indium bumps during reflow. The content after the “*” represents the optimization method.

Reflow with the assistance of UBM facilitates the shaping and adjustment of indium bumps, resulting in smooth, regular, and densely packed spherical formations due to the surface tension [149,258]. Compared to molten Sn, which has a surface tension of 407 mN/m, molten indium exhibits higher surface tension at 560 mN/m, making it particularly suitable for reflow and ball formation [224]. During reflow, molten indium naturally aligns itself with UBM, even if the patterned openings of indium bumps are initially misaligned (Figure 12f). This automatic alignment capability improves process tolerance and practicality significantly [224,259]. In ambient air environments, indium bumps do not achieve good wetting with UBM due to oxidation [260]. Therefore, controlled atmospheres consisting of inert gas, flux gas, and reducing gas are typically used during reflow soldering to prevent oxidation and ensure optimal wetting [208]. Strict control over gas composition, temperature, humidity, and duration is essential for the successful reflow process [218]. Figure 12 illustrates common optimization methods for indium bumps. The following three points should be noted: (I) Thermal reduction in a reducing gas atmosphere minimizes indium loss but may involve unacceptable reaction temperatures. For instance, high-temperature (above 380 °C) reduction in a hydrogen atmosphere can produce easily removable hydroxides, hydrides, or H_2_O, and it carries the risk of repeated oxidation and adverse effects on the performance of SWIR FPA sensors [250,256]. (II) Acid bath treatment prior to reflow effectively removes oxides but lacks controllability, leaving residual acid and causing isotropic etching that can lead to indium loss and the detachment of indium bumps [256,257]. Chemical fluxes pose residue issues, particularly in fine-pitch applications. Prolonged residue can corrode indium bumps, UBM, and surface passivation, even impeding capillary action and underfill material flow. Therefore, a prompt and thorough cleaning of indium bumps after flux use is essential [224].

Greer et al. conducted a comparative analysis of plasma and wet chemical methods for indium oxide removal, proposing a two-step plasma treatment using Ar/CH_4_/H_2_ and Ar/H_2_ [256]. This approach effectively eliminates the indium oxide without causing isotropic corrosion or undercut of indium bumps, as observed with HCl wet etching. Ar aids in plasma ignition and enhances physical bombardment on the material, playing a critical role in etching and indium oxide removal. Huang et al. utilized a Ar/CH_4_/H_2_/SF_6_ mixed gas plasma etching process to remove surface-oxidized indium [250]. Comparably to the unetched sample (42.2%), high-resolution O1s X-ray Photoelectron Spectroscopy (XPS) analysis revealed that the percentage of the In-O bond strength remained nearly unchanged at 44.5% after Ar/CH_4_/H_2_ plasma etching. The introduction of SF_6_ dramatically reduced the percentage of the In-O bond strength to 10.8%. SF_6_ showed a better oxide removal effect and prevented indium from re-oxidation. It is expected that after SF_6_-assisted plasma etching, indium oxide can be converted into oxyfluoride, to prevent the inward diffusion of oxygen [261].

**Figure 13:**
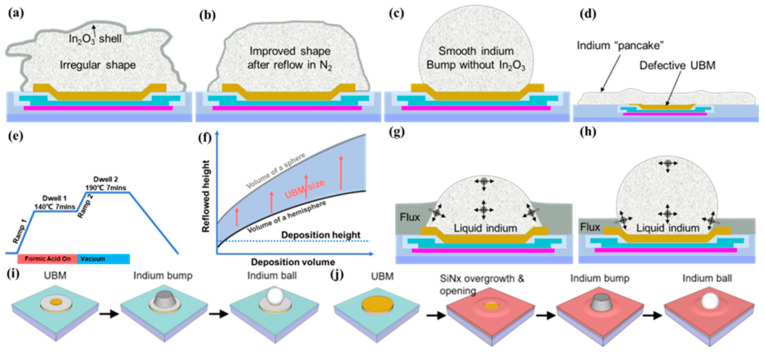
(**a**) The indium bumps before reflow in irregular shapes; (**b**) the indium bumps after reflow in N_2_; (**c**) the normally reformed indium bumps after reflow in N_2_ enriched with formic acid; (**d**) indium “pancake” on defective UBM; (**e**) the schematic two-step reflow profile; (**f**) the change in height of the indium bumps with respect to a sphere or hemisphere of the same volume [230]; (**g**) schematic diagram of surface tension of molten indium affected by a solid; and (**h**) soft fluxes [224]. The two process flow charts for the fabrication of the indium bumps [259]: (**i**) indium directly deposited on the UBM contact pads; (**j**) indium deposited on SiN_x_ opening holes on top of the UBM contact pads, reprinted with permission from ref. [259]. Copyright 2020 SPIE.

Cui et al. investigated the interaction between UBM and indium during reflow [203]. Initially, the indium bumps exhibited an irregular shape (Figure 13a). After reflow in a N_2_ atmosphere (Figure 13b), the shape of the bumps improved, but the surface remained not uniform. Figure 13c demonstrated the reflow of indium bumps in a mixture of N_2_ and a gaseous formic acid atmosphere. Due to surface tension, the indium bumps became spherical on UBM. However, defects in UBM acting as a non-wetting layer hindered the formation of smooth, spherical indium in the molten state, resulting in outward indium flow and the formation of a rough, pancake-shaped surface (Figure 13d). Therefore, a defect-free UBM is crucial for achieving smooth indium bumps post-reflow.

Jordan et al. found that the final height of reflowed indium bumps was primarily influenced by the deposited indium volume, while the diameter of the UBM of Ti/Ni plays a minor role after a two-step reflow process [230]. Firstly, surface indium oxide was removed by heating the samples below the indium melting point in a formic acid atmosphere. Subsequently, the chamber was imposed to vacuum and the temperature was raised above the melting point to initiate the reflow, resulting in uniformly smooth bumps (Figure 13e). Generally, smaller UBM diameters led to taller reflowed bumps, a trend that was clearly observed in SEM images, as depicted in Figure 13f.

Zhu et al. proposed a flux-assisted wet reflow process for indium bump arrays, with a focus on the comparing effect of flux viscosity on the sphericity of indium bumps during reflow [224] (Figure 13g,h). The use of low-viscosity liquid flux minimally disrupts the surface tension of liquid indium and balances its partial surface tension, resulting in higher sphericity compared to that of solid flux. The liquid flux immerses the indium during reflow, effectively removing the indium oxide while also acting as a barrier to oxygen. In this case, the reflow self-alignment between indium bumps and UBM was confirmed, thereby facilitating the formation of high-quality micro-bump arrays with minimal equipment requirements and stable processes.

Kozłowski et al. developed a reliable pre-reflow technique for indium bump arrays that involves wet etching and annealing in a formic acid vapor atmosphere [218]. Initially, the indium evaporation process was optimized to ensure a smoother surface and increase the indium atom density, thereby reducing oxidation within the bumps. Pre-etching annealing was employed to solve the indium bump detachment issues caused by undercuts during a subsequent 10% HCl bath treatment. Shear tests conducted on the samples demonstrated enhanced adhesion between the annealed indium bumps and UBM.

Ma et al. introduced a thin SiN_x_ layer on the UBM composed of Cr/Ni/Au and successfully achieved spherical indium bumps arrays with a 10 μm pitch [259]. Figure 13i illustrates the conventional process of directly depositing indium bumps on the UBM and then reflowing them into spherical shapes. In contrast, Figure 13j depicts the process with the SiN_x_ layer. The SiN_x_ layer is grown and then selectively etched to expose the UBM underneath [37]. The combination of UBM and the SiN_x_ layer effectively mitigates the non-uniform reflow effects of indium bumps. This optimized process offers a novel method for optimizing indium bumps and holds great promise for achieving even smaller pitch (≤10 μm).

### 3.4. Underfill

Underfill plays a pivotal role in flip-chip bonding technology by enhancing mechanical strength, mitigating stress from thermal cycling, improving thermal management, offering environmental protection, ensuring electrical isolation, and facilitating process compatibility. Together, these attributes significantly contribute to the overall reliability, performance, and longevity of epitaxial SWIR image sensors.

#### 3.4.1. Overview of Underfill Materials

Underfill is closely associated with IBM’s flip-chip and Controlled Collapse Chip Connection (C4) technologies [262,263,264]. During the early stage of flip-chip development, high-cost ceramic substrates were preferred over low-cost organic substrates [144,265,266]. This is because the ceramic substrates (coefficient of thermal expansion (CTE) for alumina ceramics is 6.9 ppm K^−1^, and CTE for Si-based chips is 2.5 ppm K^−1^) provided better mitigation against thermal cycle degradation of C4 bumps [267] (which have a CTE of 22–25 ppm K^−1^) compared to that of organic substrates (e.g., CTE for FR-4 is 18–24 ppm K^−1^) [268]. In 1987, Hitachi company proposed that the underfill technique could be used for flip-chip assembly, indicating that photosensitive resin (curable resin) is a feasible adhesive to match the CTE of the solder at the chip–substrate interface [268,269]. During the thermal cycling treatment, the underfill redistributed the thermal mechanical stress [270]. Upon curing, underfill materials provide mechanical protection to solder bumps, solving issues such as vibration, drop, and impact-induced mechanical damage [270,271,272]. They also create a barrier between the interconnect area and the environment, which was used to protect against the moisture and oxidizing agents [267,273]. Underfill materials ensure strong adhesion between the chip and substrate, thereby enhancing the reliability of the flip chip. Suitable underfill materials can reduce the strain on solder bumps by 10–25%, thereby extending the fatigue life of solder joints [267,268,274]. In this way, underfill serves as both a remedy and an insurance measure to enhance the inherent reliability of flip-chip technology.

Generally, underfill materials are composite materials with multifunctional properties, including electrical, thermal, and mechanical characteristics. They are typically composed of suspension liquid and fillers. Epoxy resin is commonly used as a suspension liquid due to its excellent chemical resistance, strong adhesion, low cost, and outstanding electrical and physical properties [275]. The type, shape, size, and surface morphology of the fillers significantly influence various properties of underfill materials, including the CTE, thermal conductivity, electrical conductivity, and viscosity [276,277,278]. For instance, pure cured epoxy resin exhibits a higher CTE (55–75 ppm K^−1^) [279]. Therefore, it is a common practice to incorporate a certain mass fraction of nano-sized silica dioxide filler (which has a CTE of around 0.5 ppm K^−1^) to create a composite material with a lower CTE [280]. To ensure an optimal flow and uniform distribution, the size of the fillers should be smaller than one-third of the fill gap [268]. The ratio of suspension liquid to fillers is crucial for balancing the flowability and functionality.

The curing process of underfill materials further influences these properties by transforming the epoxy resin into a solid crosslinked structure, which significantly enhances its mechanical strength, thermal stability, and adhesive properties. Although fillers are essential in modifying the thermal and mechanical properties of underfill materials, their incorporation during the curing process introduces several challenges. Upon curing, epoxy resin forms a crosslinked network with a disordered amorphous chain structure. This leads to significant phonon scattering within the material [281,282], which reduces the thermal conductivity of traditional epoxy resins to below 0.2 W m^−1^ K^−1^ [267]. To overcome this limitation, fillers with high intrinsic thermal conductivity, such as nano-sized metals, carbon nanotubes, and graphene, are commonly added. However, these fillers must be carefully balanced with their impact on electrical conductivity [283,284,285,286,287]. Moreover, maintaining a uniform suspension of the fillers throughout the curing process remains a challenge [288,289]. During the curing process, the filler content distribution may become not uniform along the Z-direction of the filling gap, leading to performance variations, such as changes in glass transition temperature (T_g_), CTE, toughness, and adhesion [290,291].

In light of these challenges, Wen et al. have worked on the selection criteria for ideal bottom-filling materials, focusing on attributes such as low viscosity (<20 Pa·s at 298 K), high thermal conductivity (>1.0 Wm^−1^ K^−1^), appropriate CTE (25–30 ppm K^−1^), high electrical resistivity (>10^12^ Ω·cm), low dielectric constant (<4.0 at 298 K and 1 kHz), and low dielectric loss factor [267,292,293,294] (<0.005 at 298 K and 1 kHz). However, achieving underfill materials that satisfy all these criteria remains a significant challenge; for example, an improvement in one aspect of performance often requires compromises in others. In addition to optimizing fillers, the precise molecular design or modification of epoxy resin with specific functional groups can also lead to developing underfill materials with enhanced composite functionalities [295,296].

#### 3.4.2. Underfill for SWIR FPAs

For underfill materials used in SWIR FPA applications, several basic requirements should be considered, as outlined below: (I) excellent electrical insulation and high electrical resistivity are essential to prevent short circuits between indium bumps; (II) a low dielectric constant is required to reduce parasitic capacitance between indium bumps and bonding interfaces; (III) high thermal conductivity ensures that SWIR FPAs operate within a stable temperature range; (IV) the coefficient of thermal expansion (TEC) of the underfill material should match the TECs of indium bumps in the vertical direction, as well as the TECs between SWIR FPAs and ROICs; (V) reasonable curing time is necessary to avoid affecting the underfill throughput; (VI) proper viscosity is crucial to ensure the full effect of capillary action.

##### Capillary Underfill

**Figure 14:**
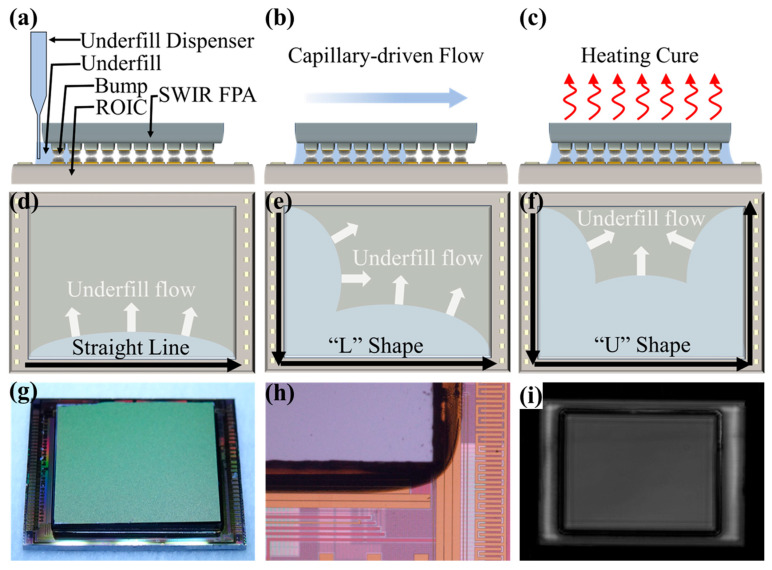
(**a**) SWIR FPA bare module dispensing diagram; (**b**) underfill material flows under capillary action; (**c**) heating cure after dispensing; (**d**) dispensing in a straight line; (**e**) dispensing in an “L” shape; (**f**) dispensing in a “U” shape; (**g**) SWIR FPA bare module after dispensing; (**h**) wetting situation of the underfill material; (**i**) ultrasonic scanning microscopy image of the IRFPA bare module.

Capillary underfill is extensively utilized in SWIR FPAs due to its high filling rate, reduced void formation, and compatibility with a wide range of underfill materials without compromising the yield of indium bumps. The specific process of capillary underfill is illustrated in Figure 14. In this process, the bonded SWIR FPA bare module is fixed on a level carrier. Prior to dispensing, preheating may be conducted to ensure the SWIR FPA remains dry and to enhance the flowability of the underfill materials. When dispensing, the apex of the SWIR FPA is used as a reference point, and the needle position is slightly elevated above the plane of the ROIC, which allows the underfill material to smoothly reach the ROIC surface while preventing mechanical damage to the SWIR FPA (Figure 14a). Additionally, the needle position should also maintain an appropriate horizontal distance from the SWIR FPA edge. Due to the imbalance between surface tension and gravity, underfill material can readily self-flow horizontally into the gap between the ROIC and SWIR FPA. Through capillary action, the underfill material progressively spreads inward, displacing the air and completely occupying the area outside the indium bumps (Figure 14b). After dispensing, the SWIR FPA is typically transferred to a vacuum oven for further processing (Figure 14c). The vacuum enhances the capillary-driven flow, mitigating or preventing void defects and non-uniformity. The oven is then heated to the curing temperature to ensure a robust indium bumps bond between the SWIR FPA and ROIC.

Three common dispensing methods (Figure 14d–f) are employed [297]: (I) dispensing in a straight line along the long side of the SWIR FPA or the side without pads of the ROIC; (II) dispensing in an “L” shape along any two adjacent sides of the SWIR FPA; and (III) dispensing in a “U” shape continuously along three sides, leaving the remaining long side untreated. In all three methods, the pads of the ROIC cannot be covered by underfill material to avoid affecting the subsequent wire bonding [298]. Following dispensing, the underfill material needs sufficient time to flow to the opposite side. Generally, a “rectangle” closed trajectory for dispensing on all four sides is not utilized since it limits the escape route for air and may result in underfill failure [268]. The duration of the entire flow filling process depends on the viscosity of the underfill materials, the density and height of the indium bumps, and the size of the SWIR FPA [299]. For the indium bump fabrication process using solder flux, the residue of the solder flux and cleaning agent cannot be ignored [300]. Figure 14g displays the SWIR FPA (320 × 256, pitch 30 μm) after “L” shape dispensing twice and the heating cure. Figure 14h shows the wetting situation in the lower right corner. Figure 14i presents the ultrasonic scanning microscopy image of the SWIR FPA under complete underfill [301,302,303].

The continuously shrinking pixel pitch in SWIR FPAs, the decreasing height of indium bumps, and the narrowing gap between the SWIR FPA and ROIC imply that they are approaching the limit of capillary underfill’s ability. Therefore, it is urgent to introduce new technologies to ensure that capillary underfill can accommodate higher-density packaging. The following methods may enhance or assist in capillary underfill: (I) Provide a vacuum-enhanced dispensing environment, such as a sealed chamber equipped with a vacuum pump. Simultaneously, adjust and modify the underfill materials to adapt to low pressure and enhance their flowability, enabling easier penetration into narrow capillary structures [236,304]. (II) Adopt a temperature gradient as a driving force for thermocapillary action to accelerate the flow of underfill material [305]. (III) Utilize the vertical underfill to handle the issue of incomplete filling caused by the rapid flow of underfill material around the edges of the chip during horizontal capillary filling [306,307], especially for large-size FPA chips (≥40 mm × 40 mm) [308].

### 3.5. Back Thinning

Flip-chip-bonded SWIR FPAs are designed to absorb infrared radiation through backside incidence [309,310,311,312]. This structure inevitably leads to reflection, absorption, and scattering, resulting in a reduced infrared flux reaching the absorption layer [313,314,315,316]. The SWIR-sensitive material requires substrate (bulk material) support and is processed into a SWIR FPA sensor using semiconductor fabrication techniques [317,318,319,320]. Notably, substrate materials, such as, CdZnTe, GaAs, InAs, GaSb, InP, etc., are opaque to SWIR to varying degrees and require thinning or removal to accommodate back-incidence after underfill, thereby minimizing radiation losses [321,322,323,324]. Through retaining a thin layer of the FPA on the ROIC, maximized QE, improved responsivity, and extensions can be obtained [325]. Specific partial substrate removal is also helpful for minimizing the spectral crosstalk between adjacent pixels [313]. To reduce reflection at the incident interface, common treatments include polishing the incident surface or applying anti-reflection coatings [326,327,328]. As the size of SWIR FPAs increases, proper back thinning can effectively alleviate thermal stress issues during rapid cooling [173,329].

The back thinning process of SWIR FPAs typically involves grinding, chemical mechanical polishing (CMP) (fast thinning) [330,331], and wet chemical etching (slow thinning) [332,333,334]. The rapid rotation of the grinding disc generates continuous mechanical friction between surface particles and the back of SWIR FPAs, resulting in scratches and cracks [335]. Under the force of gravity from the fixture and ceramic tray, these overlapping damage layers continuously expand downwards, thinning the SWIR FPA to limit the thickness achievable by grinding [336]. CMP is necessary to eliminate the surface defects and microcracks caused by grinding and achieve further thinning [337], involving physical and chemical reactions [338,339]. Surface roughness can be further reduced using smaller abrasives. After CMP, the SWIR FPA thickness must be maintained within a certain range to prevent damage and ensure structural reliability in subsequent steps, necessitating the limitation of the CMP process [214]. Moreover, the mesh-like underfill in the FPA-ROIC gap offers substantial support to areas without indium bumps, especially in the state of extremely thin absorption layer and volume shrinkage of the underfill [340,341]. Wet etching allows the substrate thickness to be reduced to sub-micron dimensions without physical damage [334].

**Figure 15:**
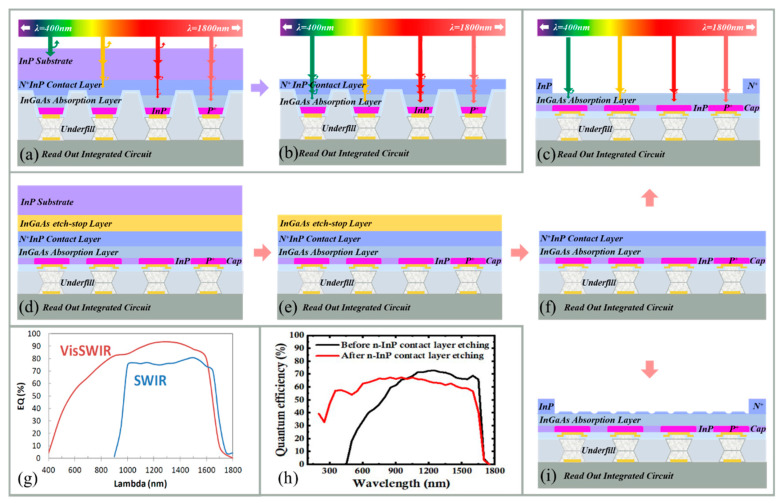
Backside illuminated InGaAs/InP SWIR-FPA visible extension schematic principle. (**a**) Backside-illuminated mesa-type InGaAs/InP SWIR-FPA; (**b**) after InP substrate removal; (**c**) retained N-type InP contact layer around the pixel array; (**d**) backside-illuminated planar-type InGaAs/InP SWIR-FPA with etch-stop layer; (**e**) after InP substrate removal; (**f**) after InGaAs etch-stop layer removal; (**g**) measured QE on SWIR and Vis-SWIR FPA [154], reprinted with permission from ref. [154], Copyright 2013 SPIE; (**h**) QE before and after n-InP contact layer etching; (**i**) 10 nm left N-type InP contact layer [350], reproduced from [350], open access by MDPI, 2024. The arrows under the spectra in (**a**–**c**) indicate the absorption and reflection of different bands of infrared radiation for the InGaAs/InP SWIR-FPA.

By thinning the backside and removing the InP substrate, the response spectrum of backside-illuminated InGaAs/InP PIN SWIR FPAs can be extended into the near-infrared and even the visible light range (Figure 15a,b) [74,342,343,344]. The cross-section of the mesa-type InGaAs/InP SWIR FPA is illustrated in Figure 15a, and it primarily divides into three layers from top to bottom: sensor epitaxial structure, indium bumps encapsulated by underfill, and ROIC. The common epitaxial structure encompasses a N-type InP contact layer, an InGaAs absorption layer, and an InP cap layer, which is suitable to both planar and mesa types [310,345,346]. The N-type InP substrate (InP bandgap width is 1.35 eV, and absorption cutoff wavelength is 920 nm) exhibits a high absorption coefficient for visible light [74,347]. Consequently, only short-wavelength infrared can penetrate InP and reach the InGaAs layer [315] (In_0.53_Ga_0.47_As bandgap width is 0.75 eV, and absorption cutoff wavelength is 1700 nm). However, reflection and absorption within the material cannot be entirely avoided during the incident process [319,348,349].

Rouvié et al. have demonstrated a study where the InP substrate has been completely removed from a planar InGaAs/InP SWIR FPA. In this structure, the surface of the N-type InP contact layer was coated with an anti-reflection film composed of SiO_2_/TiO_2_ [351,352] adapted for the Vis-SWIR range (achieving a reflectance coefficient of less than 6% within the wavelength range of 400–1700 nm) [154]. As a result, the vis-SWIR FPA achieved a QE of 40% at 500 nm, 75% at 800 nm, and over 80% between 900 nm and 1600 nm (Figure 15g). He et al. utilized an epitaxial structure incorporating an InGaAs etch-stop layer (Figure 15d) [334]. The removal of the InP substrate consisted of two stages (Figure 15e): Initially, thinning to 100 μm was conducted through mechanical polishing, followed by etching with a mixture of phosphoric acid and hydrochloric acid [353,354,355]. Tartaric acid exhibited excellent etching selectivity [356,357,358] for the InGaAs and N-type InP contact layer, enabling the removal of the InGaAs etch-stop layer while protecting the N-type InP contact layer (Figure 15f). Subsequently, Inductively Coupled Plasma (ICP) etching [359,360,361] was performed to thin the N-type InP contact layer to 10 nm (Figure 15i), resulting in a vis-extended InGaAs/InP FPA with a QE exceeding 60% in the range of 500–1700 nm. The reflectance of the N-type InP contact layer surface after plasma etching could be reduced to as low as 17% [334,362]. Zhang et al. further etched the N-type InP contact layer, retaining only the portion surrounding the pixel array (Figure 15c), thereby extending the spectral response to 200–1700 nm. The QE exceeded 45% in the range of 300–1650 nm (Figure 15h) [350].

## 4. Summary and Outlook

The research and development of high-resolution, cost-effective epitaxial SWIR image sensors with advanced performances has a long and rich history. Currently, InGaAs acts as an ideal absorber for SWIR image sensors due to its relatively mature growth techniques, versatile composition control, and wavelength coverage within the SWIR range. With the increasing demand for the SWIR imaging market, it is anticipated that the price of epitaxial InGaAs SWIR image sensors will gradually decline. However, the cost of these sensors has previously been constrained by the limited availability of larger InP substrates and expensive process flows. To greatly decrease the price for epitaxial SWIR image sensors, it is significantly important to identify a CMOS-compatible SWIR absorber. To this end, group IV Ge (Sn) materials have entered the researcher’s field of vision, which is primarily attributed to their CMOS compatibility, ability to grow epitaxially on Si substrates, excellent photoresponse in the SWIR band, and adjustable bandgap (through strain engineering, alloy engineering, and doping engineering). To compete with the InGaAs SWIR image sensor, the GOI (Germanium-on-Insulator) structure has emerged as a timely innovation. Furthermore, to extend the spectral response range of Ge SWIR image sensors (which typically have a cutoff-wavelength of 1.7 μm), GeSn image sensors feature e-SWIR properties, suggesting GeSn image sensors are one of the promising technical routines to compete with e-SWIR InGaAs image sensors.

In the meantime, although significant progress has been made over the past two decades, developing high-resolution epitaxial SWIR image sensors, such as those with a smaller pixel pitch (<10 μm), a smaller pixel size (<5 μm), and miniaturized FPAs (>2560 × 2048), has been mainly limited by flip-chip bump bonding technology. Even though Sony Company has introduced Cu-Cu flip-chip bonding technology (with a pixel pitch of 5 μm), the specifications of the resulting FPAs are still at least an order of magnitude lower than that of CMOS sensors. In the future, bolstered by Cu-Cu bonding, epitaxial SWIR image sensors will possess enhanced dimensional miniaturization potential, potentially attaining an integration level and pixel pitch comparable to those of CISs. This holds particular significance for the CMOS-compatible Ge (Sn) SWIR image sensor, which anticipates being fabricated entirely using CIS processes. Nevertheless, the verification of the feasibility and reliability of Cu-Cu bonding in the context of epitaxial SWIR image sensors demands substantial experimental substantiation, and the path of research and development still stretches far into the distance. First, flip-chip bump bonding technology remains the predominant method for integrating SWIR image sensors into commercial applications, which was crucial in achieving high-resolution SWIR sensors as well as meeting the increasing demand for enhanced imaging capabilities across various industries. The development of more advanced flip-chip technologies is eagerly anticipated to further improve the performance and functionality of SWIR sensors. Second, the flexibility of flip-chip technology is particularly advantageous when integrating novel epitaxial SWIR materials. Epitaxy plays a critical role in semiconductor manufacturing by enabling precise control over the crystalline structure and composition of materials. Moreover, flip-chip technology supports the ongoing miniaturization of SWIR sensors, a trend driven by the demand for compact, lightweight, and portable imaging devices. By reducing the size and footprint of sensor components, flip-chip bonding facilitates the development of high-performance SWIR cameras. Furthermore, the compatibility of flip-chip bonding with CMOS processes enhances the manufacturability and cost-effectiveness of SWIR sensor production, which offers the advantages of scalability, low power consumption, high integration density, performance optimization, and enhanced functionality of SWIR imaging systems. With the continued development of InGaAs and Ge (Sn) material growth technologies and flip-chip bump bonding technology, we believe that high-resolution FPAs with specifications comparable to those of CMOS sensors can be expected in the future. Breakthroughs in epitaxial SWIR image sensors will open up new research avenues and lead to broad applications in life sciences, medical diagnostics, defense, surveillance, security, free-space optics (FSO), thermography, agriculture, food inspection, LiDAR, and beyond.

## Figures and Tables

**Table 1:** Reported epitaxial InGaAs SWIR FPAs with spectral response range of 0.9–3.0 μm, in terms of active region composition, growth tools, resolution, pitch, pixel size, wafer size, and the measured dark current and quantum efficiency.

Year	Active Region	Growth Tools	Resolution	Pitch	Pixel Size	Wafer Size	Dark Current	Quantum Efficiency	Ref
2005	In_0.53_Ga_0.47_As	MOCVD	640 × 512	25 μm	——	<100 mm	<1 pA	85%@1310 nm 80%@1550 nm	[74]
2009	In_0.53_Ga_0.47_As	MOCVD	1280 × 1024	15 μm	——	<100 mm	——	——	[75]
2014	In_0.53_Ga_0.47_As	MBE	512 × 128	30 μm	23 μm	<100 mm	35 nA/cm^2^	——	[71]
2014	In_0.83_Ga_0.17_As	MBE	640 × 512	25 μm	20 μm	<100 mm		>30%@1310 nm >70%@1550 nm	[72]
2014	In_0.53_Ga_0.47_As	——	640 × 512	——	25 μm	——	≤0.2 pA	≥70%	[76]
2014	In_0.53_Ga_0.47_As	MOVPE	1024 ×1280	12.5 μm	——	100 mm	0.7 nA/cm^2^	>80%	[77]
2021	In_0.75_Ga_0.25_As	MBE	320 × 256	24 μm	≤20 μm	<100 mm	5.2 nA/cm^2^	58%	[73]
	In_0.83_Ga_0.17_As			30 μm			21 nA/cm^2^	57.4%	
2021	In_0.53_Ga_0.47_As	MOCVD	1280 × 1024	10 μm	15 μm	100 mm	2.25 nA/cm^2^	80%@1550 nm	[78]
2022	In_0.83_Ga_0.17_As	MBE	1280 × 1024	——	15 μm	<100 mm	133 nA/cm^2^	69%	[66]
2022	In_0.53_Ga_0.47_As	MBE	2560 × 2048	10 μm	——	<100 mm	3.34 nA/cm^2^	73.7%	[79]

**Table 2:** Reported epitaxial Ge (Sn) SWIR FPAs with spectral response range of 0.9–1.7 μm, in terms of active region, growth tools, resolution, pitch, pixel size, wafer size, and the measured dark current and quantum efficiency.

Year	Active Region	Growth Tools	Resolution	Pitch	Pixel Size	Wafer Size	Dark Current	Quantum Efficiency	Ref
2008	Ge	CVD	128 × 128	7 µm	10 µm	200 mm	——	30%@1310 nm	[127]
2009	Ge	CVD	64 × 8	——	——	——	25 mA/cm^2^	——	[128]
2010	Ge	CVD	640 × 480	10 µm	10 × 10 µm^2^	200 mm	25 fA	44%@1310 nm	[129]
2011	Ge	CVD	64 × 64	150 µm	——	——	4.1 × 10^−5^ A/cm^2^	30%@1500 nm	[130]
2016	GeSn	MBE	320 × 256	30 µm	——	<200 mm	10^−6^ A/cm^2^	——	[131]
2017	Ge	CVD	——	——	——	300 mm	0.93 μA	——	[132]
2021	Ge	MBE	64 × 1	25 µm	20 µm	——	100 pA	——	[133]
2022	Ge	MBE	320 × 256	30 µm	27 × 27 µm^2^	100 mm	9.7 nA	48.9%@1550 nm	[134]

**Table 3:** UBM options for indium bump system, in terms of year, institution, UBM stack layers (thickness), and deposition method.

Year	Institution	UBM Stack Layers (Thickness)	Deposition Method	Ref
2004	Northwestern University	Ti/Pt/Au (NA)	Electron Beam Evaporation	[202]
2006	Paul Scherrer Institute	Ti/Ni/Au (10/50/50 nm)	Sputtering	[152]
2009	Shanghai Institute of Microsystem and Information Technology, Chinese Academy of Sciences	Ti/Pt/Au (20/30/80 nm)	Sputtering	[231]
2013	Hong Kong University of Science and Technology	Ti/Ni/Au (20/50/50 nm)	Electron Beam Evaporation	[227]
2018	Fraunhofer Institute for Reliability and Microintegration, IZM, Technical University Berlin	Ni/Au (NA)	Sputtering	[232]
2019	i3system Inc	Ti/Au (NA)	Evaporation	[228]
2020	Northwestern University	Ti/Ni/Au (20/30/100 nm)	Evaporation	[229]
2021	Sandia National Laboratories	Ti/Ni (NA)	Electron Beam Evaporation	[230]
2022	Institute of Semiconductors, Chinese Academy of Sciences	Ti/Pt/Au (NA)	NA	[134]
2023	Device Technology Research Institute (Japan)	Ti/Au (10/30 nm)	Physical Vapor Deposition	[233]
